# Correlation of neuroimaging and thorax CT findings in patients with COVID-19: A large single-center experience

**DOI:** 10.3906/sag-2105-138

**Published:** 2021-08-29

**Authors:** Hediye Pınar GÜNBEY, Günay RONA, Özge ADIGÜZEL KARAOYSAL, Ayşe BATIREL, Banu ÖZEN BARUT

**Affiliations:** 1 Department of Radiology, Faculty of Medicine, University of Health Sciences Kartal Lutfi Kırdar City Hospital, İstanbul, Turkey; 2 Department of Infectious Diseases, Faculty of Medicine, University of Health Sciences Kartal Lutfi Kırdar City Hospital, İstanbul, Turkey; 3 Department of Neurology, Faculty of Medicine, University of Health Sciences Kartal Lutfi Kırdar City Hospital, İstanbul, Turkey

**Keywords:** COVID-19, brain, CT, MR

## Abstract

**Background/aim:**

The aim of this current study was to describe the neuroimaging findings among patients with COVID-19 and to compare them with thorax CT imaging findings and clinicobiological profiles.

**Materials and methods:**

Between the period March 11 and December 31, 2020, we evaluated brain computed tomography (CT) and magnetic resonance (MR) images of patients with COVID-19. A total of 354 patients (mean age 65.2 ± 16.6, 52% female, 42% male) who had brain imaging were included in the study. Of this total sample, 218 had thorax CT scanning (65.5%). Neuroimaging and thorax CT findings, clinical course, neurologic findings, and laboratory data were evaluated. White matter lesions (WML) and thorax CT scans were scored. Participants were divided according to whether or not they had an infarction.

**Results:**

The neuroimaging findings indicated infarcts, parenchymal hemorrhage, encephalitis, cortical signal abnormality, posterior reversible encephalopathy syndrome (PRES), and cranial nerve involvement. WML significantly positively correlated with age (p < 0.01) but not with sex (p > 0.05). Thorax CT findings did not demonstrate significant correlations with infarcts, WML, or hemorrhages (p > 0.05). D-dimer and ferritin levels were significantly higher among patients with infarcts (p < 0.05).

**Conclusion:**

Immune-mediated prothrombic state and cytokine storm appear to be more responsible for etiopathogenesis than direct viral neurotropism. Neuroimaging and thorax CT findings were not correlated among patients with COVID-19 in our study. These results suggest that neurological manifestations may occur independently of pulmonary involvement and age.

## 1. Introduction

Coronavirus disease (COVID-19) caused by the novel coronavirus severe acute respiratory syndrome— Coronavirus-2 (SARS-CoV-2) was first reported in December 2019 in Wuhan, China. Its rapid spread worldwide has caused more than 119,000,000 infections and nearly more than 2,600,000 deaths (as of March 15, 2021). The most commonly reported CT findings in patients with COVID-19 pneumonia were bilateral, ground-glass opacity in the lower lobes, consolidation, and a “crazy-paving” pattern [1]. In addition to systemic and respiratory symptoms of other coronaviruses (CoVs), the neurologic involvement of SARS-CoV-2 has been discussed in more detail in recent literature [2–5]. For example, common manifestations have been described as headache, dizziness, nausea, ischemic stroke, intracerebral hemorrhage, epilepsy, myelitis, Guillain–Barré syndrome, Bell’s palsy, and rhabdomyolysis with decreasing frequency [6,7]. From initial case reports, COVID-19-associated acute hemorrhagic necrotizing encephalopathy [8], encephalitis/meningitis [9–11], hemorrhagic PRES [12,13], acute disseminated encephalomyelitis (ADEM) [14,15], cerebral venous thrombosis [16–18], and acute ischemic stroke [10,18,19] have also been reported as other neuroimaging findings. Most neuroimaging publications about COVID-19 are case reports/series, with few large patients’ series. The aim of this current study was to describe neuroimaging findings among patients with COVID-19 and compare them with thorax CT imaging findings and clinicobiological profiles.

## 2. Materials and methods

### 2.1. Study design and patient cohort

During the period March 11 and December 31, 2020, we evaluated brain CT and magnetic resonance (MR) images of patients with COVID-19. Local institutional review board approval was obtained from local ethics committees and the Ministry of Health Scientific Research Platform for this retrospective study. The requirement for written consent from patients was waived in accordance with the Council for International Organizations of Medical Sciences guidelines. The inclusion criteria were: i) diagnosis of COVID-19 infection using reverse-transcriptase–polymerase-chain-reaction (RT-PCR) or by chest CT showing results consistent with SARS-CoV-2-associated pneumonia, ii) existence of acute neurological symptoms, iii) age greater than 18 years, and iv) availability for brain MRI or CT. The exclusion criteria were the presence of underlying CNS disease, including stroke and trauma. The neuroimaging and thorax CT findings, clinical course, neurologic findings, and laboratory data were acquired from the hospital information system and picture archiving communication systems (PACS) of our institution. The D_dimer, C-reactive protein, and ferritin levels were noted as suspected prognostic laboratory tests for COVID-19.

### 2.2. Imaging protocols

Patients were scanned using a 1.5T MRI scanner (Philips Ingenia^®^) with a 20-channel head-coil (Philips^®^, the Netherlands). The brain MRI protocol included axial T1 weighted (W) conventional spin-echo sequence with and without gadolinium [repetition time (TR)/echo time (TE): 450/15 ms, slice thickness: 5 mm, field of view (FOV): 230, matrix: 256 × 163; number of excitations (NEX): 2, intersection gap: 1 mm, scanning time: 2 min 30 s]; axial T2W turbo spin-echo sequence [TR/TE: 4443/100 ms, section thickness: 5 mm, FOV: 230, matrix: 384 × 240, NEX: 2, intersection gap: 1 mm, scanning time: 3 min 37 s], axial and coronal fluid attenuation inversion recovery [TR/TE/TI: 8000/140/2800 ms, section thickness: 5 mm, FOV: 230, matrix: 224 × 148, NEX: 2, intersection gap: 0.4 mm, scanning time: 2 min 40 s], sagittal T2W turbo spin-echo sequence [TR/TE: 4027/100 ms, section thickness: 5 mm, FOV: 230, matrix: 236 × 205, NEX: 2, intersection gap: 1 mm, scanning time: 1 min 05 s], and contrast-enhanced axial, coronal, and sagittal T1W conventional spin-echo sequence [TR/TE: 461/10 ms; section thickness: 5 mm, FOV: 230, matrix: 240 × 166, NEX: 2, intersection gap: 0.89 mm, scanning time: 1 min 43 s]. Diffusion-weighted imaging was performed with a single-shot spin-echo planar image (ss-EPI) [TR/TE: 3,376/74 ms; section thickness: 5 mm; FOV: 230 mm; matrix: 128 × 128; NEX: 2; intersection gap: 1 mm; b values: 0 and 1,000 s/mm2]. Imaging parameters for susceptibility weighted imaging were as follows: TR/TE, 26/50 ms; FA, 20°; slice thickness, 2 mm; slice gap; voxel size 0.9 × 1.06 × 2 mm, 130 slices; and scanning time: 3′ and 55″. Where encephalitis was suspected, dynamic susceptibility contrast-enhanced (DSE) perfusion and MR spectroscopy were acquired. Brain and thorax CT scans were obtained using a 128-slice Philips Ingenuity Multidetector CT (Philips Medical Systems, Best, the Netherlands). The thorax CT parameters were, respectively, as follows: tube voltage, 100–120 kVp; tube current, standard (reference mAs; 200–50); slice thickness, 5.0–5.0 mm; pitch 1.558–1.293. Scans were taken in the supine position during normal respiration. No intravenous contrast agent was used. The axial acquisition non-contrast head CT protocol was as follows: tube voltage, 135 kVp; tube current, standard (reference mAs; 200–50); with a standard 512 × 512 matrix; and 24 cm FOV at 5.0 mm section thickness.

### 2.3. Image analysis

Brain MRI and CT scans were initially analyzed by a neuroradiologist (H.P.G) with 10 years’ experience and reviewed for evidence of ischemic infarcts, intracerebral micro/macro-hemorrhage, edema, venous thrombosis, cerebral atrophy, PRES, parenchymal and meningeal enhancement, multifocal white matter lesions (WML), white and gray matter FLAIR signal abnormalities, and cranial nerve abnormalities. WML were classified as mild, moderate, or severe. Participants were divided into two groups: patients with infarcts (PWI), including with acute/ subacute infarct, and those with any infarct (non-PWI). Additional findings were noted.

The thorax CT scans were analyzed by a radiologist (G.R) with six years’ experience of thoracic imaging at our institution. Evaluation identified ground glass density, consolidation, and “crazy paving.” These lesions were scored according to the area of involvement in the lung. Scoring was performed using the semi-quantitative method recommended by Pan et al. [20]. Accordingly, the lesions in all five lobes of the lung were scored visually between 0 and 5 (where 0 = no involvement, 1 = less than 5% involvement, 2 = 5%–25% involvement, 3 = 26%–49% involvement, 4 = 50%–75% involvement, and 5 = more than 75% involvement). For each patient, a value in the range of 0–25 was obtained from the sum of all lobes. Accordingly, definitions were: mild = 1–9 points, moderate = 10–19 points, and severe = 20–25 points. No involvement (0) was considered normal. The radiologists were blinded from each other while evaluating thorax CT and brain MRI/CT scans.

### 2.4. Statistical analysis

Statistical analyses were performed using SPSS version 17.0. Descriptive statistics include the mean (SD) as median (min–max) or as frequency (%). The Kolmogorov–Smirnov test was used to analyze the normal distribution assumption for quantitative outcomes. Due to normally distributed data, to identify the difference between values, we used the Student t test for normally distributed data. Categorical data variables were compared using either Pearson’s chi-squared test or Fisher’s exact test. The correlation coefficient values range from +1 to −1, where +1 indicates a perfect positive relationship, −1 indicates a perfect negative relationship, and a 0 indicates no relationship exists. If the value is near ± 1, then it said to be a perfect, between ± 0.50 and ± 1, strong, between ± 0.30 and ± 0.49 medium, below + .29 small correlation.

## 3. Results

### 3.1. Patient characteristics and clinical data

A total of 381 patients who had brain imaging with a diagnosis of COVID-19 were recruited to the study, and 27 patients (7%) were subsequently excluded following application of the exclusion criteria. Of the remaining 354 patients (93%) who met the inclusion criteria, 103 (29%) had brain MRI [61 female (59%), 42 male (41%)] and 254 had brain CT imaging [132 female (51.9%), 122 male (48.1%)]. The mean age of patients who had brain MRI was 64.4 ± 18.6 years and 69.2 ± 15.3 years for those who had brain CT. The abnormality detection rate was 4.7% for CT and 28% for MR imaging. The most common neurologic symptoms were headache (57%), fever (16%), syncope (13%), weakness (7%), gait disturbance (4%), and altered mental status (3%). Of the 354 patients who had neuroimaging, 28 died (6.4%) with accompanying comorbid factors, such as diabetes mellitus (DM), hypertension (HT), coronary artery disease (CAD), demans, and epilepsy (in descending order). Neuro-imaging diagnosis rates for patients who died are shown in Table 1.

### 3.2. Neuro-imaging findings

The findings from brain MRI, CT, and diffusion images revealed infarcts, parenchymal hemorrhages, encephalitis, cortical signal abnormality, PRES, and cranial nerve involvement. Brain and diffusion MRI rates are shown in Table 2. According to MRI findings (n = 103 total), the WML classification ratios were as follows: none 31% (n = 32), mild 16.9% (n = 17), moderate 19.7% (n = 20), and severe 32.4% (n =34) in patients.

#### 3.2.1. Infarcts

The ratio of infarcts was 17.5% in total (female = 18.3%, male 16.6%). The most commonly affected localizations were the cortical/subcortical area, cerebellum, brain stem, thalamus/ basal ganglia, watershed zones, and the middle cerebral artery (MCA)/posterior cerebral artery (PCA) feeding areas. The distribution of infarcts is shown in Table 3.

**Figure 1:**
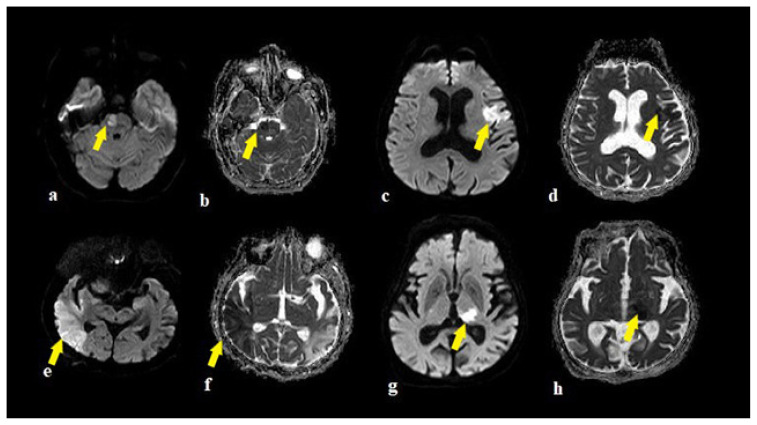
The examples of infarctions in a 64 years old female in pons (a,b), in a 68 years old man in Broca region (c,d), in 43 years old man right MCA territory (e,f), in a 32 years old man in left talamus (g,h) on DWI and ADC images (arrows).

There was no significant difference according to sex, age, and WML between participants in the PWI and nonPWI groups (p > 0.05) (Table 4). While male patients with infarction demonstrated either a moderate or severe degree of WML, infarctions among female patients were evident in all degrees of severity. The examples of brain stem infarctions are presented in Figures 1a and 1b, and cerebral infarctions are presented in Figures 1c,1d, 1e, 1f, 1g, 1h.

#### 3.2.2. Parenchymal hemorrhage

**Figure 2:**
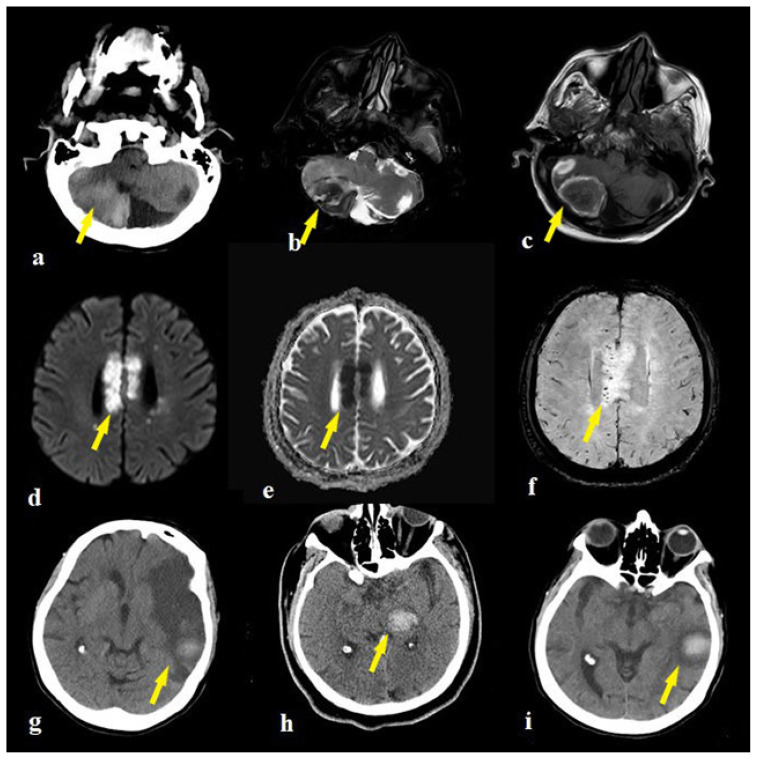
The right cerebellar hemorrhage appearences of a 74 years old man on CT (a) and axial T2W (b), T1W (c) MR images (arrows). The corpus callosum infarct of a 74 years old man on DWI/ADC (d,e) and microhemorrhages on SWI images (f) (arrows). The macrohemorrhages of a 64 years old female in the left MCA infarction zones (g,i) and a 46 years old man in the left thalamus (h) on CT (arrows).

Intraparenchymal hemorrhage was identified as macro and micro hemorrhages in the cerebellar hemisphere (Figure 2a, 2b, 2c) and MCA territory (Figure 2f, 2g, 2h, 2i), in infarcts. All patients who demonstrated hemorrhage or hemorrhagic transformation had DM, HT, and CAD as comorbid factors.

#### 3.2.3. Encephalitis

**Figure 3:**
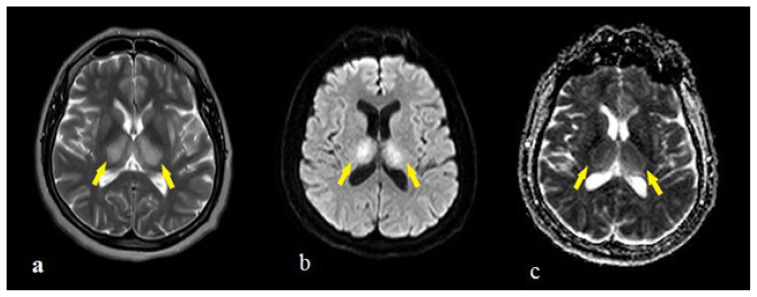
MRI appearences of a 54 years old female patient show T2 hyperintensity with expansion (a) and diffusion restriction (b,c) in bilateral thalamus and slightly in bilateral putamen (arrows).

COVID-19-associated encephalitis with a fatal course was seen in two patients. The first patient was a 54-year-old female patient admitted to the emergency department with complaints of respiratory distress and altered mental state. She had T2/FLAIR hyperintensity with expansion (Figure 3a) and diffusion restriction (Figure 3b, 3c) in the bilateral thalamus and slightly in the bilateral putamen. In the follow-up process, the patient had generalized tonic-clonic seizures and was intubated. Additional neuroradiological scanning could not be completed, and the patient died after 11 days.

**Figure 4:**
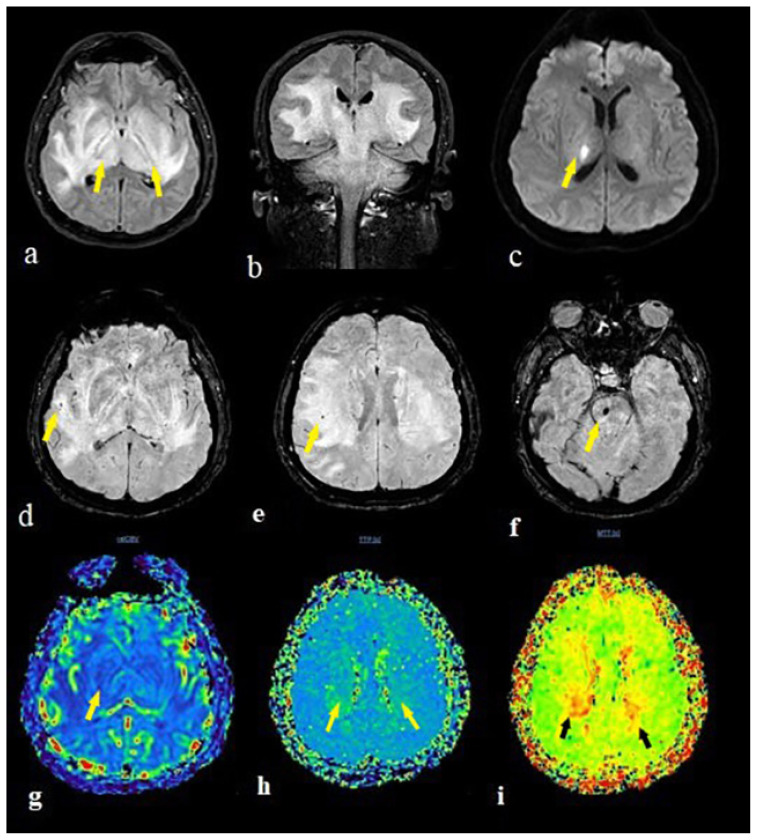
The brain MRI of a 41 years old male patient with encephalitis showed extensive hypertens signal in bilateral cerebral hemisphere white matter, thalamus, basal ganglia corticospinal tract, brain stem and serebellum on FLAIR images (a,b). The diffusion restrction occured only in the right thalamus on DWI sequence (c). There were microhemorrhages in the pons(f) and right parietal deep white matter (d,e) on SWI images. The perfusion MR imaging showed any CBV increment (g) with prolongation in bilateral thalamus and right temporal lobe on time to peak (h) and mean transit time (i) maps (arrows).

The second patient was a 49-year-old male patient admitted to the emergency department with complaints of headache, vertigo, and imbalance. He was diagnosed with DMandCOVID-19diseaseonemonthpreviously. Inthefirst brain MRI, there were T2/FLAIR hyperintense edematous signal abnormalities without diffusion restriction in the right thalamus, basal ganglia, corticospinal tract, and right side of the brain stem. During the clinical course, the patient worsened and was intubated. In the follow-up imaging, the signal abnormalities extended to bilateral cerebral hemisphere white matter, the thalamus, basal ganglia corticospinal tract, brain stem, and cerebellum (Figures 4a, 4b). Diffusion restriction occurred in the right thalamus (Figure 4c). There were micro hemorrhages in the pons and right parietal deep white matter (Figures 4d, 4e, 4f). Perfusion MR imaging highlighted showed any CBV increment (Figure 4g) and prolongation of time to peak (Figure 4h) and mean transit time maps (Figure 4i) in the bilateral thalamus and right temporal lobe. The patient died four days after initial admission to hospital, with death recorded as hemorrhagic necrotizing encephalitis.

#### 3.2.4. Posterior reversible encephalopathy syndrome

**Figure 5:**
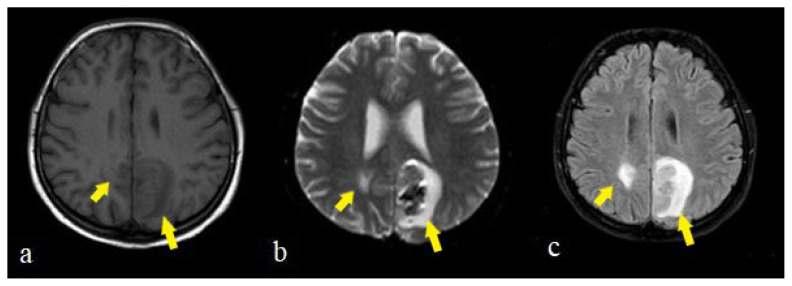
The brain MRI of a 28 years old female patient showed bilateral parieto occipital cortical hypointensity on T1W (a), hyperintensity on T2W (b), and FLAIR (c) images with hemorrhagic transfromation on the left (b) (arrows).

The patient with a diagnosis of PRES was a 28-year-old COVID-19-positive female patient with complaints of headache, vertigo, and seizure. She also had kidney transplant history. Brain imaging revealed bilateral parietal occipital cortical T2/FLAIR hyperintensity with prominent diffusion restriction and hemorrhagic transformation seen on the left side (Figure 5a, 5b, 5c).

#### 3.2.5. Cranial nerve involvement

**Figure 6:**
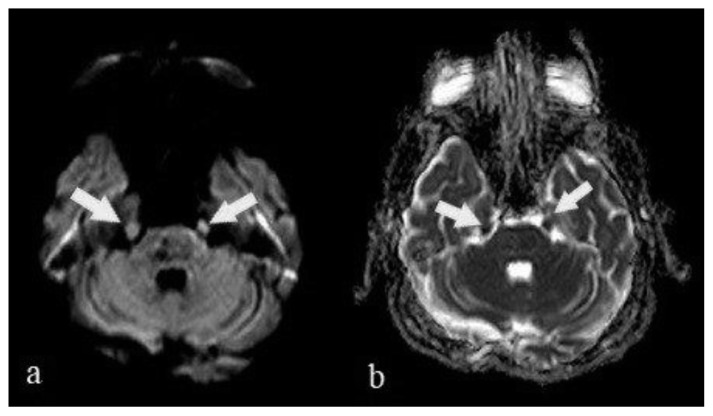
Bilateral trigeminal nerve thickening of a 69 years old man was revealed on DWI (a) and ADC (b) images (arrows).

Bilateral trigeminal nerve thickening was revealed by diffusion MRI in a 67-year-old man with severe COVID-19 pneumonia. Additional cranial nerve involvement and contrast enhancement could not be revealed due to the poor clinical course and intubation of the patient (Figure 6a, 6b).

### 3.3. Thorax CT findings

Of 354 patients who had neuroimaging, 218 had thorax CT scanning (65.5%). Ground glass density, consolidation, and crazy paving were noted in the thorax CT evaluation. Scoring of these lesions was performed using the semiquantitative method recommended by Pan et al., as described in the Methods section. Findings such as chronic airway disease (not defined as a COVID-19-related disease) were not included in the scoring. According to scoring, the distribution of thorax CT findings were normal 24.9% (n = 54), mild 33.2% (n = 72), moderate 17.5% (n = 38), severe 10.6% (n = 23), and 13.8% (n = 31) who were nonCOVID.

### 3.4. Correlations

There was an expected significant correlation between the WML score and age (r = 0.493, p = 0.001). The WML score showed no significant correlation with sex (p = 0.076). Thorax CT findings did not achieve significant correlations with infarcts (p = 0.081), WML (p = 0.121), or hemorrhages (p = 0.96) (Table 5). One patient who died from encephalitis had mild pneumonic infiltration. D-dimer and ferritin levels were significantly higher in the PWI group compared with the non-PWI group (p = 0.034). The D-dimer, CRP, and ferritin levels showed a positive correlation with each other in the PWI group (r = 0.482, p = 0.01), and the D-dimer levels were significantly higher for males than for females (p = 0.037).

## 4. Discussion

While the general clinical presentation and respiratory results were speculated aspects of COVID-19, various neurological manifestations have also been reported during the course of the disease. The evidence of this retrospective neuroimaging study demonstrates the association of CNS involvement with SARS-CoV-2 infection. According to COVID-19 neuro-imaging reports, the current study (with a relatively large patient number from a single major center) has similar patient characteristics in terms of age and sex [21]. For the distribution of patients who had neuroimaging and then subsequently died, those without any pathology ranked first, while those with infarction ranked second. The most common comorbidities were DM, HT, and CAD among patients who died from COVID-19 (due to the exclusion criteria, we did not investigate patients with chronic neurological disorders).

The most common finding here was that non-specific imaging findings were compatible with existing literature [22]. Ischemic infarcts were diagnosed in 28% of our patients, which is similar to findings reported by Coughar and colleagues [23]. Using MRI in the diagnosis of infarction may have increased our percentage according to the literature. The distribution of arterial infarcts included small vessel, acute large vessel, and watershed infarcts. We did not describe any venous infarcts or venous sinus thromboses in our patient cohort. Coagulopathy is a well-described contributing factor, leading to both arterial and venous thromboembolic events in patients with COVID-19 [24]. Therefore, the significant level of inflammatory markers (such as D-dimer, CRP, and ferritin levels) confirms this pathway in stroke patients included in our study. However, it is not clear whether hyper coagulopathy is related to endotheliopathy by direct viral infection or from an abnormal inflammatory response. In our study, the rate of infarction was not correlated with age, WML, and pneumonic infiltration, suggesting endothelial damage rather than inflammation. Accompanying comorbidities such as DM, HT, and CAD may have contributed to this process with an increased risk of vasculopathy.

Intracranial hemorrhage in different intracranial compartments is a common neurologic complication of COVID-19. Coagulopathies, increased blood pressure, anticoagulation therapy, disseminated intravascular coagulation, and cytokine storm with underlying endoteliopathy may cause micro and macro hemorrhaging. Patients may present with lobar hemorrhage, subarachnoid/ subdural hemorrhage, or microhemorrhages. Microhemorrhages may also present as hemorrhagic transformation of infarcts, as in our patients.

The inflammatory reaction of SARS-CoV-2 with cytokine storm damages the blood brain barrier and results in increased vascular permeability in PRES patients. Patients present with complaints of uncertain symptoms such as headaches, seizures, and altered consciousness. Brain MRI typically reveals bilaterally vasogenic edema in the parietal and occipital regions, with occasionally accompanying hemorrhagic components [25]. Beside coagulopathy, the reported acute kidney injury among COVID-19 patients may lead to rapid changes in blood pressure, cerebral edema, microembolization, and microhemorrhages. Our patient with a PRES diagnosis also had renal transplantation history, which compromises this suggestion.

Encephalitis is a less common but more critical type of neuronal involvement of COVID-19. Besides clinical and laboratory examination, imaging is a powerful tool for the diagnosis and follow-up of the disease. Described encephalitis cases present a myriad of radiological findings and a few reports indicating brain stem and thalamus involvement, as in our case, which have been reported previously [8,26,27]. While they may not have been routinely confirmed by histopathology, the possible suggested mechanisms present direct viral neuro-invasion or intracranial cytokine storm and blood-brain barrier damage.

Cranial nerve involvement involving the olfactory bulb, optic nerve, abducens nerve, and facial nerve have been reported previously [28]. To our knowledge, this is the first presentation of trigeminal nerve involvement in COVID-19. The involvement of other cranial nerves and nuclei (except the olfactory nerve) suggests invasion beyond the olfactory bulb in the course of the disease. Moreover, according to some authors, it may be related to axonal spread and viral replication leading to inflammation and demyelination [29,30]. The inability to detect SARS-CoV-2 in the CSF until today means the immunomediated responses may also be responsible for cranial neuropathies.

To our knowledge, there is a lack of sufficient data in the literature to demonstrate the link between COVID-19 neuroimaging and thorax CT findings to date. In our study, the severity of neuroimaging findings in infarcts and encephalitis did not correlate with the severity of thorax CT findings. Some patients had neurological complaints in the presence of normal thorax CT findings and any pulmonary symptoms. These findings suggest a delayed immune-mediated process underlying the physiopathology of CNS damage regardless of respiratory involvement [31].

CNS involvement by the virus and subsequent immune response may be the causes of neuropathology that occurs with the disease. The hematogenous dissemination of the virus with infected leukocytes through compromised endothelial cells of the blood-brain barrier (referred to as a Trojan horse) is the first mechanism of CNS migration [32]. The other strongly suggested possible migration mechanism is the peripheral invasion of the virus through the olfactory bulb or vagus nerve, which, thereafter, spreads to different zones of the brain. The infectious potential of SARS-CoV-2 is based on using angiotensin-converting enzyme 2 (ACE2) pathways to enter cells, as with other coronaviruses. ACE2 receptors are expressed in the epithelia of the lung and small intestine and also in the brain (including neurons, astrocytes, oligodendrocytes, substantia nigra, ventricles, middle temporal gyrus, posterior cingulate cortex, and olfactory bulb) [32,33]. Furthermore, the immune-mediated process that causes cytokine storm is the other underlying neuropathological pathway of CNS damage observed in COVID-19 [34].

Our study has some limitations. First, it is a retrospective study, and we did not investigate the patients with chronic neurological disease where COVID-19 may worsen the current prognosis. The PCR analysis of COVID-19 from cerebrospinal fluid could not be evaluated due to a lack of lab kits. Finally, we could not represent an interobserver difference in our study.

In conclusion, the increasing frequency of neuromanifestations of COVID-19 appears in many forms, including infarcts, WML, PRES, encephalitis, and cranial nerve involvements. However, this study shows that the neuroimaging findings do not correlate with age and thorax CT findings. It seems that neurological involvement tends to increase in the presence of co-morbid factors such as HT, DM, and CAD. Although the exact etiopathogenesis is scant, the immune-mediated prothrombic state and cytokine storm appear to be more responsible than direct viral neurotropism. Neuroimaging plays a critical role in determining neurological complications with significant mortality and morbidity during the COVID-19 pandemic process. Further advanced neuroimaging studies with diffusion tensor imaging and functional MRI may provide information on the long-term effects of COVID-19 on the microstructures of the brain.

## Figures and Tables

**Table 1:** Neroimaging diagnosis rates of the patients who died of COVID-19.

Neuroimaging findings	Percentage
Encephalitis	**4%**
Cranial nerve involvement	**5%**
Infarct	**41%**
Nonspesific	**50%**

**Table 2:** Brain and diffusion MRI findings rates of COVID-19 patients.

Brain and diffusion MRI findings	Percentage
Normal	**63%**
Infarct	**28%**
Hemorrhage	**4%**
Encephalitis	**2%**
PRES	**1%**
Cranial nerve involvement	**1%**
Cortical signal abnormality	**1%**

**Table 3:** The distrubution of occured infarcts.

The distrubution of infarcts	Percentage
Cortical-subcortical	**42%**
Watersheed zones	**8%**
MCA-PCA feding zones	**4%**
Thalamus-basal ganglia	**20%**
Brain stem	**12%**
Cerebellum	**12%**
Corpus callosum	**2%**

MCA: middle cerebral artery, PCA: posterior cerebreal artery.

**Table 4:** There was no significant difference according to sex, WML, and positive thorax CT findings between participants in the PWI and nonPWI groups (p > 0.05).

		Levene’s Test for Equality of Variances		t-test for Equality of Means						
									95% Confidence Interval of the Difference	
		F	Sig.	t	df	Sig. (2-tailed)	Mean Difference	Std. Error Difference	Lower	Upper
**sex**	Equal variances assumed	.457	**.500**	.325	215	.746	.029	.090	−.148	.206
	Equal variances not assumed			.323	53.548	.748	.029	.090	−.152	.210
**WML**	Equal variances assumed	.550	**.461**	.925	69	.358	.28522	.30847	−.33016	.90060
	Equal variances not assumed			.936	51.168	.354	.28522	.30471	−.32646	.89689
**thorax_CT**	Equal variances assumed	.063	**.802**	1.199	215	.232	.28668	.23903	−.18447	.75783
	Equal variances not assumed			1.185	53.226	.241	.28668	.24193	−.19852	.77188

**Table 5:** Correlation of neuroimaging findings with age/sex and thorax CT findings.

Correlated data	*p* value
WML score / age	r = 0.493, *p =* 0.001
WML score / sex	r = 0.18, *p* = 0.076
Thorax CT findings / infarcts	r = 0.36, *p* = 0.081
Thorax CT findings / hemorrhages	r = 0.49, *p* = 0.96
Thorax CT findings / WML	r = 0.26, *p* = 0.121

WML: White matter lesions.
